# Virulence factors of Shiga toxin-producing *Escherichia coli* and the risk of developing haemolytic uraemic syndrome in Norway, 1992–2013

**DOI:** 10.1007/s10096-017-2974-z

**Published:** 2017-04-08

**Authors:** U. Naseer, I. Løbersli, M. Hindrum, T. Bruvik, L. T. Brandal

**Affiliations:** 1 Domain for Environmental Health and Infectious Disease Control, Norwegian Institute of Public Health, P.O. Box 4404 Nydalen, 0403 Oslo, Norway; 2 European Programme for Public Health Microbiology Training (EUPHEM), European Centre for Disease Prevention and Control (ECDC), Stockholm, Sweden; 3 Nextera AS, Oslo, Norway

## Abstract

Shiga toxin-producing *Escherichia coli* (STEC) may cause haemolytic uraemic syndrome (HUS). Age ≤5 years and presence of *stx2a* and *eae* are risk factors for the development of HUS. In this study, we investigated STEC isolates for the presence of adhesins, toxins and molecular risk assessment (MRA) factors to identify virulence genes associated with HUS development. We included non-duplicate isolates from all STEC infections (*n* = 340, HUS = 32) reported to the Norwegian National Reference Laboratory (NRL) for Enteropathogenic Bacteria from 1992 to 2013. The most common STEC were O157:H7/H^−^ (34%) and O103:H2 (14%). We retrospectively screened the isolates by three multiplex polymerase chain reactions (PCRs) for adhesins (*n* = 11), toxins (*n* = 5) and MRA (*n* = 15). We calculated odds ratios (ORs) and adjusted odds ratios (aORs) for associations with HUS development. On average, isolates were positive for 15 virulence genes (range: 1–24); two toxins (range: 0–4), five adhesins (range: 0–8) and eight MRA genes (range: 0–13). The gene combinations were clustered within serotypes. Isolates from HUS cases were positive for *eae* and *IpfA*_O26_, and negative for *saa*, *eibG*, *astA*, *cnf*, *subA* and *pic*. We identified 11 virulence genes with a significant association to HUS development. Multivariable analyses adjusted for age group and Shiga toxin identified *nleH1–2* [aOR 8.4, 95% confidence interval (CI); 2.18–32.3] as an independent risk factor for the development of HUS from an STEC infection. This study demonstrated that the non-LEE effector protein *nleH1–2* may be an important predictor for elevated risk of developing HUS from STEC infections. We recommend the NRL for Enteropathogenic Bacteria to consider including *nleH1–2* screening as part of routine STEC surveillance.

**Electronic supplementary material:**

The online version of this article (doi:10.1007/s10096-017-2974-z) contains supplementary material, which is available to authorized users.

## Introduction

Shiga toxin-producing *Escherichia coli* (STEC) is a zoonotic food- and waterborne pathogen of a serious public health concern because of its propensity to cause outbreaks, haemorrhagic colitis and the potentially life-threatening complication haemolytic uraemic syndrome (HUS) [1, 2]. It is estimated that 6–25% of patients infected with STEC develop HUS, with up to 50% requiring renal dialysis. In children, this rate is much higher [1]. HUS generally complicates 6–9% of STEC infections overall and about 15% of infections in children, with mortality of 2–5% and up to 30% developing long-term sequelae [2, 3]. Currently, there are no available treatments for HUS and prevention strategies are limited.

The production of bacteriophage-encoded Shiga toxin 2a (Stx2a) by STEC is the primary virulence trait responsible for HUS development, along with the presence of intimin (*eae*) and young age of the host (≤5 years old) [4–6]. However, not all cases of STEC infections harbouring these characteristics develop HUS. The clinical significance of STEC for humans is further determined by the production and interplay of additional virulence factors, as well as host factors such as immunocompetence [7, 8].

Typically, STEC strains harbour the genomic island termed the ‘locus of enterocyte effacement’ (LEE), which encodes genes that facilitate the colonisation process (e.g. *eae*). The LEE also encodes a series of proteins that induce cytoskeletal changes in the eukaryotic target cell to enhance attachment and effacement of the bacterium [9, 10]. In addition, many virulence factors, such as the plasmid-encoded enterohaemolysin (*ehxA*), have been shown to be important for the pathogenicity of STEC. A number of candidate pathogenicity islands (PAIs), including OI-122 and OI-71, encode a variable repertoire of non-LEE-encoded effector (*nle*) proteins which are present in many STEC strains. However, their role in disease development is not yet fully understood [8, 11, 12].

Recently, we published a comprehensive study spanning 20 years from 1992 to 2012, where we investigated host factors such as age, sex and clinical manifestations, and strain factors such as serotypes, *stx* types and the presence of *eae* and *ehxA*, with the association of developing HUS from STEC infections in Norway [5]. Following in line with the concept of a molecular risk assessment (MRA) [13], the objective of this study was to identify virulence factors important for HUS development, to enable an improved differentiation of HUS-associated STEC from low virulent STEC infections and expand the Norwegian STEC surveillance system.

## Methods

### Case definition, strain and data collection

An STEC infection was defined as the isolation of an STEC from a clinical sample. We retrospectively included all non-duplicate STEC isolates, one isolate per outbreak (unless different virulence gene profiles were detected), submitted to the National Reference Laboratory (NRL) for Enteropathogenic Bacteria from 1992 to 2012 (*n* = 334). In addition, we included all STEC isolates recovered from HUS patients in Norway in 2013 (*n* = 6). HUS was defined as acute renal failure within 14 days of an episode of acute diarrhoea with either microangiopathic haemolytic anaemia or thrombocytopaenia. Clinical and epidemiological information of cases corresponding to the selected STEC isolates were retrieved from the Norwegian Surveillance System for Communicable Diseases database.

### Serotyping

All isolates were consecutively serotyped at the NRL on reception using slide agglutination against 43 different O groups, *fliC*-PCR for H groups and *wzx*- and *wzy*-PCR for 14 O groups for non-agglutinating isolates, as described previously [5].

### Sorbitol-fermenting (SF) *E. coli* O157

Isolates belonging to serogroup O157 were analysed for sorbitol fermentation by polymerase chain reaction (PCR) as described previously [5].

### Virulence genes characterisation

All isolates were consecutively screened for the presence of genes *stx1a*, *c*, *d*, *stx2a*–*g*, *eae* and *ehxA* by PCRs, as described previously [5]. In addition, all isolates were retrospectively screened for five toxin genes (*cnf*, *astA*, *subA*, *StcE*/*StcE*_*O103*_, *cdtB I–IV*), 11 adhesin genes (*saa*, *ihaA*, *agn43*_*EDL933*_, *Ipf1*_*O157*/*OI-141*_, *IpfA*_*O26*_, *Ipf2*_*O157*/*OI-154*_, *IpfA*_*O113*_, *eibG*, *toxB*, *espP*, *ehaA*) and 15 genes previously classified as MRA genes [14–18] (*aaiC*, *fyuA*, *ent*/*espL2n*, *nleB*, *nleE*, *efa1*/*lifA*, *pagC*-like, *nleH1–2*, *ureC*, *nleC*, *ecf4*, *paa*, *pic*, *nleG2–3*, Z2099) using three multiplex PCRs. For all PCRs, DNA was extracted by boiling, primers were 5′ end labelled (6-FAM, VIC or PET), and PCRs were run and analysed as described previously (Supplemental Table 3) [19].

### Clustering and statistics

We performed cluster analysis with the presence and absence of 33 virulence genes and subtypes of *stx1* and *stx2* in BioNumerics v.7.6 (Applied Maths, Belgium) using an unweighted pair group method with arithmetic mean (UPGMA) for phylogeny. Statistical analyses were performed in Stata version 13.1 (StataCorpLP, USA). Univariable analyses were performed by calculating odds ratios (ORs) by assigning the presence of virulence genes as cases and absence thereof as controls, and HUS as the outcome variable. Virulence genes were considered significantly associated with HUS if the *p*-value was <0.05. Significant factors were analysed in a multivariable logistic regression model adjusted for age group and Shiga toxin to calculate adjusted odds ratios (aORs) with 95% confidence intervals (CIs).

## Results

### Description of cases

The description of cases (*n* = 333) and characteristics of isolates (*n* = 334) from 1992 to 2012 have been published previously [5]. Briefly, the total number of cases was 339, with 190 females (56%) and median age 14 years (range <1 to 97). The age distribution displayed two peaks, at age groups ≤5 years (*n* = 134, 40%) and 21–40 years (*n* = 67, 20%). Half of the cases (*n* = 171, 50%) were non-import cases, 146 (43%) were hospitalised and 31 (9%) were reported to have developed HUS.

### Serogroups and serotypes

A total of 340 isolates were typed into 24 different O serogroups. The most frequent O serogroups included O157 (*n* = 115, 34%), O103 (*n* = 50, 15%), O26 (*n* = 34, 10%) and O145 (*n* = 25, 7%). The most frequent serotypes included O157:H7/H^−^ (*n* = 115, 34%), O103:H2 (*n* = 47, 14%), O26:H11 (*n* = 34, 10%) and O145:H28 (*n* = 18, 5%). Ten of the O157 isolates (9%) were sorbitol fermenters.

### Distribution of *stx* genes

A total of 218 isolates (64%) were positive for *stx1* [*stx1a* (*n* = 192), *stx1c* (*n* = 23), *stx1d* (*n* = 3)], 212 isolates (62%) were positive for *stx2* [*stx2a* (*n* = 90), *stx2b* (*n* = 32), *stx2c* (*n* = 101), *stx2d* (*n* = 9), *stx2g* (*n* = 2)] and 91 isolates (43%) were positive for both *stx1* and *stx2*.

### Distribution of toxins, adhesins and MRA

On average, isolates were positive for 15 virulence genes (range: 1–24); two toxins (range: 0–4), five adhesins (range: 0–8) and eight MRA genes (range: 0–13). A total of 319 isolates (94%) were identified with toxin genes other than *stx1* and/or *stx2*. The most common toxin genes identified were *ehxA* (*n* = 290), *StcE*/*StcE*_*O103*_ (*n* = 194) and *subA* (*n* = 42). A total of 338 isolates (99%) were identified with adhesion genes, most commonly *IpfA*_*O26*_ (*n* = 306), *eae* (*n* = 252) and *ihaA* (*n* = 243). A total of 306 (90%) of the isolates were identified with at least one MRA gene. The most common genes identified were *nleB* (*n* = 250), Z2099 (*n* = 248), *nleE* and *ent*/*espL2n* (*n* = 247) (Table 1).

**Table 1:** Virulence genes present and absent in isolates from the four most frequent Shiga toxin-producing *Escherichia coli* (STEC) serotypes and associated cases of haemolytic uraemic syndrome (HUS)

Serotype	No.	Present in all	Absent from all	HUS (*n*)	Present in all HUS	Absent from all HUS
O157:H^−^	115	*eae*, *Ipf1*_*O157*/*OI-141*_, *Ipf2*_*O157*/*OI-154*_	*saa*, *aaiC*, *IpfA*_*O113*_, *ehaA*, *subA*	12	*IpfA*_*O26*_	*eibG*, *astA*, *cnf*, *fyuA*, *pic*
O103:H2	47	*IpfA*_*O26*_, *ehaA*, *efa1*/*lifA*, Z2099, *nleE*, *nleB*	*aaiC*, *toxB*, *IpfA*_*O113*_ *, Ipf2*_*O157*/*OI-154*_, *eibG*, *cnf*, *cdtB I–IV*, *nleH1–2*, *fyuA*, *pic*	0		
O26:H11	34	*eae*, *iha1*, *IpfA*_*O113*_, *IpfA*_*O26*_, *ehaA*, *efa1*/*lifA*, *ecf4*, Z2099, *nleE*, *nleB*, *paa*, *fyuA*, *ent*/*espL2n*	*saa*, *Ipf1*_*O157*/*OI-141*_, *Ipf2*_*O157*/*OI-154*_, *eibG*, *toxB*, *astA*, *cnf*, *cdtB I–IV*, *subA*, *nleC*, *pic*	4	*agn43*_*EDL933*_, *ureC*, *nleH1–2*, *nleG2–3*	*pagC*-like
O145:H28	18	*iha1*, *agn43*_*EDL933*_, *Ipf1*_*O157*/*OI-141*_, *IpfA*_*O26*_, *ehxA*, *efa1*/*lifA*, Z2099, *nleB*, *nleE*, *paa*, *ureC*, *ent*/*espL2n*	*saa*, *aaiC*, *toxB*, *IpfA*_*O113*_, *ehaA*, *Ipf2*_*O157*/*OI-154*_, *eibG*, *astA*, *cnf*, *cdtB I–IV*, *subA*, *nleH1–2*, *fyuA*, *nleG2–3*	1	*eae*, *espP*, *ecf4*, Z2009	*pagC*-like, *nleC*, *pic*

#### *eae*-positive (*n* = 252) and *eae*-negative (*n* = 88)

All *eae*-positive isolates were negative for *aaiC* and *eibG*, in addition to the majority being negative for *saa* (*n* = 251), *cnf* (*n* = 251), *subA* (*n* = 250) and *astA* (*n* = 248). All *eae*-negative isolates were also negative for *toxB* and *nleH1–2*, in addition to the majority being negative for *cnf* (*n* = 87), *nleC* (*n* = 86), *nleG2–3* (*n* = 86) and *aaiC* (*n* = 85). All HUS cases were positive for *eae*.

#### O157:H7/H^−^ (*n* = 115)

On average, O157:H7/H^−^ isolates were positive for 21 virulence genes (range: 9–24); two toxins (range: 1–4), seven adhesins (range: 4–8) and 11 MRA (range: 0–13). All O157:H7/H^−^ isolates were positive for *eae*, *Ipf1*_*O157*/*OI-141*_ and *Ipf2*_*O157*/*OI-154*_. Conversely, none were positive for *saa*, *aaiC*, *IpfA*_*O113*_, *ehaA* and *subA*. Major differences between non-sorbitol-fermenting O157 (NSFO157) and SFO157 were seen for *ihaA*, *espP*, *toxB* and *ureC*, which were absent from all SFO157 (*n* = 10) and present in almost all NSFO157 (*ihaA*, *n* = 105; *espP*, *n* = 95; *toxB*, *n* = 104; *ureC*, *n* = 101). Conversely, *cdtB I–IV* was present in the majority of SFO157 (*n* = 7, 70%) and in a minority of NSFO157 (*n* = 8, 8%). Among isolates from patients developing HUS of this serotype (*n* = 12), all isolates were positive for *IpfA*_*O26*_ and all were negative for *eibG*, *astA*, *cnf*, *fyuA* and *pic*. Differences among the NSFO157 and SFO157 developing HUS were seen for *ihaA*, *agn43*_*EDL933*_, *espP* and *toxB*, which were present in all NSFO157 and absent from all SFO157.

#### O103:H2 (*n* = 47)

On average, O103:H2 isolates were positive for 13 virulence genes (range: 11–17); two toxins (range: 1–3), three adhesins (range: 2–7) and eight MRA (range: 6–9). All O103:H2 isolates were positive for *IpfA*_*O26*_, *ehaA*, *efa1*/*lifA*, Z2099, *nleE* and *nleB*. Conversely, none of the isolates were positive for *aaiC*, *toxB*, *IpfA*_*O113*_, *Ipf2*_*O157*/*OI-154*_, *eibG*, *cnf*, *cdtB I–IV*, *nleH1–2*, *fyuA* and *pic*. All STEC O103:H2 carried *stx1a* and none of these isolates were from cases that developed HUS.

#### O26:H11 (*n* = 34)

On average, O26:H11 isolates were positive for 19 virulence genes (range: 16–20); one toxin (range: 1–2), seven adhesins (range: 6–7) and 11 MRA (range: 9–12). All O26:H11 isolates were positive for *eae*, *iha1*, *IpfA*_*O113*_, *IpfA*_*O26*_, *ehaA*, *efa1*/*lifA*, *ecf4*, Z2099, *nleE*, *nleB*, *paa*, *fyuA* and *ent*/*espL2n*, and all were negative for *saa*, *Ipf1*_*O157*/*OI-141*_, *Ipf2*_*O157*/*OI-154*_, *eibG*, *toxB*, *astA*, *cnf*, *cdtB I–IV*, *subA*, *nleC* and *pic*. Among isolates from patients developing HUS of this serotype (*n* = 4), all isolates were positive for *agn43*_*EDL933*_, *ureC*, *nleH1–2* and *nleG2–3*, and all were negative for *pagC*-like.

#### O145:H28 (*n* = 18) and O145:H? (*n* = 7)

On average, O145:H28 isolates were positive for 15 virulence genes (range: 14–16); two toxins (range: 1–2), five adhesins (range: 4–6) and eight MRA (range: 7–9). All O145:H28 isolates were positive for *iha1*, *agn43*_*EDL933*_, *Ipf1*_*O157*/*OI-141*_, *IpfA*_*O26*_, *ehxA*, *efa1*/*lifA*, Z2099, *nleB*, *nleE*, *paa*, *ureC* and *ent*/*espL2n*, and all were negative for *saa*, *aaiC*, *toxB*, *IpfA*_*O113*_, *ehaA*, *Ipf2*_*O157*/*OI-154*_, *eibG*, *astA*, *cnf*, *cdtB I–IV*, *subA*, *nleH1–2*, *fyuA* and *nleG2–3*. One of the O145:H28 isolates was recovered from a case that developed HUS and five O145:H? isolates were recovered from cases that developed HUS. All HUS isolates were positive for *stx2a*, *IpfA*_*O113*_ and *nleH1–2*, whereas all non-HUS isolates were negative. Conversely, all isolates from HUS cases were negative for *iha1*, *agn43*_*EDL933*_, *Ipf1*_*O157*/*OI-141*_ and *StcE*/*StcE*_*O103*_, whereas all non-HUS isolates were positive.

## Clusters and statistical associations

**Fig. 1:**
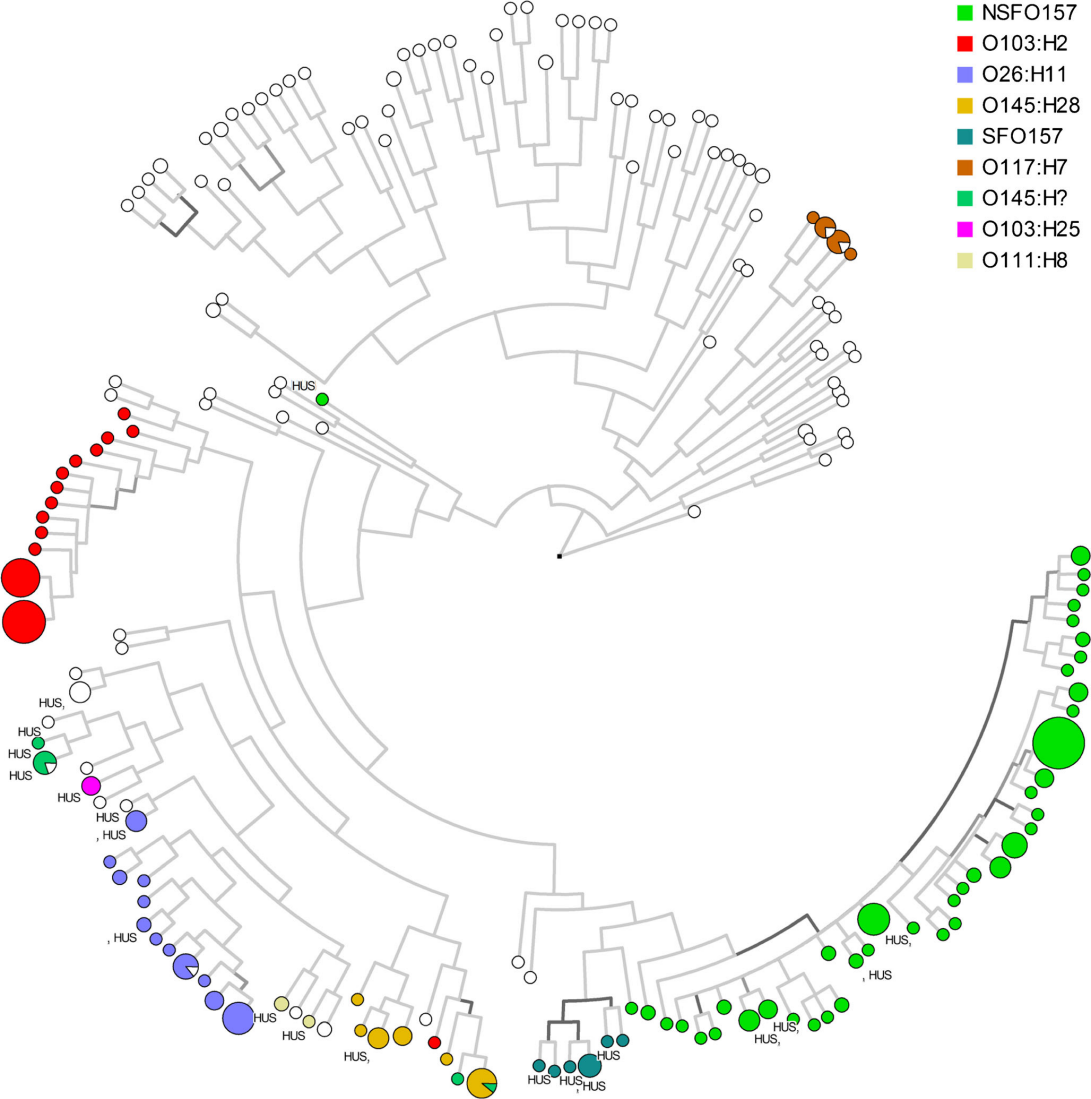
Unweighted pair group method with arithmetic mean (UPGMA) phylogenetic estimation of the relatedness of Shiga toxin-producing *Escherichia coli* (STEC) isolates (*n* = 340) based on the absence or presence of 33 virulence genes and subtypes of *stx1* and *stx2*. Phylogeny constructed using the majority summary method with 200 sample permutations. Branch intensity is according to descending resampling support (light–dark). Global cophenetic correlation was estimated to be 94%. Each node represents a unique combination of virulence genes, with the size of the node correlating to the number of isolates with that combination. Major serotypes are coloured according to the legend. Isolates from cases developing haemolytic uraemic syndrome (HUS) are indicated on the figure

Analysis based on an UPGMA phylogeny dispersed the isolates into diverse virulence gene combinations (Fig. 1). Phylogenetic clusters of related isolates were seen within the dominant serotypes, O157:H7/H^−^, O103:H2, O26:H11 and O145:H28. Isolates from cases developing HUS were seen distributed between multiple clusters, with certain clusters appearing to be less commonly associated with HUS than others. All isolates from HUS cases (*n* = 32) were positive for *eae* and *IpfA*_*O26*_, and none were positive for *saa*, *eibG*, *astA*, *cnf*, *subA* and *pic*. None of the isolates recovered from cases aged between 20 and 60 years (*n* = 117), were serotyped as O103:H2 (*n* = 47), positive for *stx2b* (*n* = 32), *stx2d* (*n* = 3), *stx2g* (*n* = 2), *stx1c* (*n* = 23) or *stx1d* (*n* = 3), or recovered from cases that developed HUS. Univariable analyses identified 11 virulence genes with a significant association with the development of HUS in addition to age ≤5 years and *stx2a* (Table 2). Multivariable analyses independent of serotype, when adjusted for age group and presence of Stx, confirmed age ≤5 years (aOR 12.7, 95% CI; 4.2–39), *stx2a* (aOR 28.6, 95% CI; 12.7–158) and the virulence gene *nleH1–2* (aOR 8.4, 95% CI; 2.18–32.3) as independent risk factors for the development of HUS (Table 2).

**Table 2:** Factors in STEC isolates with a significant (*p* < 0.05) association to the development of HUS (*n* = 32) among STEC infections in Norway 1992–2013 (*n* = 340)

Factor	Cases exposed		Controls exposed		OR^a^	*p*-Value	aOR	95% CI^b^
	*n*	%	*n*	%				
*stx2a*	29	90.6	61	19.8	39.1	<0.001	28.6	7.83–104
age ≤5 years^c^	27	84.4	108	35.1	10.0	<0.001	12.7	4.16–39.0
*efa1*/*lifA*	31	96.9	211	68.5	14.3	<0.01		
*paa*	31	96.9	211	68.5	14.3	<0.01		
*ecf4*	31	96.9	215	69.9	13.4	<0.01		
*nleE*	31	96.9	216	70.1	13.2	<0.01		
*ent*/*espL2n*	31	96.9	216	70.1	13.2	<0.01		
*nleB*	31	96.9	219	71.1	12.6	<0.01		
*nleH1–2*	29	90.6	135	43.8	12.4	<0.001	8.40	2.18–32.3
*pagC*-like	26	81.3	152	49.4	4.45	<0.01		
Z2099	29	90.6	219	71.1	3.39	<0.05		
*nleG2–3*	24	75.0	152	49.4	3.08	<0.01		
*lpfA*_O113_	19	59.4	103	33.4	2.91	<0.01		

^a^ *OR* odds ratio

^b^ *aOR*, *95% CI* adjusted odds ratio with 95% confidence interval

^c^Host factor

## Discussion

Age (≤5 years) and the presence of an *stx2a*- and *eae*-positive STEC have previously been identified as risk factors for the development of HUS in several studies [4, 20–25]. In Norway, these associations were confirmed in a recent study which estimated the odds of developing HUS to be 16 times greater for children aged 5 years or less (OR 16.7) and 30 times greater for infections with *stx2a*-producing STEC (OR 30.1) [5]. In the present study, we investigated the presence of 31 different virulence genes; five toxin genes, 12 adhesion genes and 14 MRA-associated genes, in addition to *eae*, *ehxA* and subtypes of *stx1* and *stx2*, and their association with HUS development.

Our results showed that the distribution and combination of virulence genes were clustered within distinct serotypes, with cases of HUS dispersed among the different virulent gene clusters. The largest accumulation and combination of virulence genes was seen among isolates of serotype O157:H^−^/H7. The combination of virulence genes differed between NSFO157 and SFO157. We observed, as demonstrated in previous studies, that the mosaic structure of OI-43 in SFO157 isolates lacked the *iha1* and *ureC* genes [26]. Also, as seen in earlier studies, the plasmid pO157-associated genes *espA* and *toxB* were absent from our SFO157 isolates [27]. The NSFO157 isolates were mostly negative for cytolethal distending toxin gene *cdt* (8%) compared to SFO157 isolates, which were typically positive (70%). An association of *cdt* and HUS among *eae*-negative non-SFO157 has previously been suggested, although the association between *cdt* and HUS in SFO157 is still unclear [28, 29]. Our results showed that 67% of SFO157 isolates associated with HUS were *cdt*-positive. Also, however, SFO157 isolates not associated with HUS were positive for *cdt* (75%). In addition to sorbitol fermentation, the presence of *stx2a* was the marker with the highest positive predictive value (PPV) for O157 association with HUS (29%). A single NSFO157 isolate carried only an *stx2c* subtype of Stx and was associated with HUS. This isolate displayed a very different virulence gene profile to the other NSFO157 isolates, and was the only isolate negative for *nleC*, *ent*/*espL2n*, *pagC*-like, *nleG2–3*, *nleH1–2*, *ureC*, *paa*, *nleB*, *ec4*, *efa1*/*lifA* and *StcE*/*StcE*_*O103*_ (Fig. 1). Its virulence gene combination suggested a low virulent strain and it was supposed that unknown host factors may have played an important role in the development of HUS in this case. This was supported by the fact that this STEC was isolated from a 66-year old-patient, who was the only HUS patient above 12 years of age in the study population.

The least number and combination of virulence genes among the most frequent serotypes were observed for O103:H2 isolates. Most O103:H2 isolates were grouped within two major virulence gene clusters, separated only by the presence and absence of *agn43*_*EDL933*_ and *nleG2–3*. *nleG2–3* is encoded on the pathogenicity island OI-57, which also encodes Z2099. As previously shown, all O103:H2 isolates were positive for Z2099 [30], but only one of the two clusters was positive for *nleG2–3*. Although the function of OI-57 is not entirely clear, OI-57 has previously been demonstrated to be significantly associated with human pathogenic STEC [8, 30, 31]. All O103:H25 isolates in our collection were associated with HUS and positive for *stx2a*, *IpfA*_*O113*_ and *nleH1–2*, whereas all O103:H2 isolates were negative for these virulence factors. Furthermore, only two of the O103:H2 isolates but all of the O103:H25 isolates (*n* = 3) were positive for *ureC* and *pagC*-like [32].

Among the virulence genes screened, five were encoded on the pathogenicity island OI-122 (*efa1*/*lifA*, *ent*/*espL2n*, *nleB*, *nleE*, *pagC*-like). A complete OI-122 was seen in 98% of the O157 isolates, with only two isolates negative for *efa1*/*lifA*. Among the O103:H2 isolates, OI-122 genes *efa1*/*lifA*, *ent*/*espL2n*, *nle* and *nleE* were seen to be co-located in 98% of the isolates, with *pagC*-like being only present in two isolates. An absent or truncated *pagC* may be an indicator for low virulence, as shown earlier [33]. The absence of *pagC*-like is also evident in O26:H11 isolates, all of which were positive for all other OI-122-associated genes. However, contrary to O103 isolates, all O26:H11 isolates that were associated with HUS were negative for *pagC*-like. In O26:H11 isolates, as seen for O157 isolates, *stx2a* was the marker with the highest PPV for association with HUS (44%). Isolates of the O145 serogroup with an H? phenotype were more frequently associated with HUS than H28 (71% vs. 5%). Overall, our results indicated that the virulence gene composition varies within each serotype, along with the combination of virulence genes required for an HUS-associated subtype (Supplemental Fig. 2).

In our univariable analysis, all of the OI-57 and OI-122 genes together with *cdt*, *IpfA*_*O113*_ and *ureC*, the plasmid-encoded *ecf4* (pO157) and the pathogenicity island OI-71 located in *nleH1–2* were seen to be significantly associated with the development of HUS. However, in a multivariable logistic regression model adjusted for age and *stx2a*, only *nleH1–2* remained with a significant independent association with HUS (aOR 8.4). *nleH1–2* has previously been described as an immune system modulator, functioning through inhibition of the NF-KB activation [34, 35]. Studies have proposed that it likely exhibits a role in the colonisation process rather than the attachment and effacement phase of an STEC infection [36]. Its location on OI-71 has been suggested to be an important discriminator, along with OI-122 for highly virulent enteropathogenic *Escherichia coli* (EPEC) and STEC strains [8, 13, 18, 37].

The sensitivity of *nleH1–2* in the detection of STEC isolates recovered from HUS patients was estimated to be 91%, which implied that only three isolates recovered from HUS patients were negative for *nleH1–2*. Included in these isolates was the *stx2c*-only NSFO157 isolate from a 66-year-old patient and an O111 isolate recovered from an HUS patient with multi-strain infection where one strain was *nleH1–2*-positive (carrying *stx1a* and *stx2a*) and the other strain was negative (*stx1a* only). Excluding these two isolates as probable non-HUS-associated, the sensitivity of *nleH1–2* increased to 97%, matching the sensitivity of *stx2a*, although with a lower specificity (56% vs. 80%). The negative predictive value (NPV), when combining age (≤5 years), *eae*, *stx2a* and *nleH1–2*, was estimated to be 97%, indicating that the likelihood of developing HUS was very low if all these factors were negative. The PPV was estimated to be 73%, which was an increase from 68% if we only considered age (≤5 years), *eae* and *stx2a*. Also, the specificity increased from 96 to 97% when including *nleH1–2*, allowing for a more accurate exclusion of non-HUS-associated STEC. The matter of concern was the low sensitivity (75%), which implied that 25% of the STEC isolated from HUS patients in the current study did not harbour this combination of risk factors. However, when excluding the two probable non-HUS-associated isolates, the sensitivity increased to 80%. Overall, the inclusion of *nleH1–2* increased our probability of discerning HUS-associated STEC, although other virulence factors and host-specific factors are important when assessing patients at risk of developing HUS.

Our study was limited by the virulence genes selected and the number of isolates tested. A methodological selection bias was present for O157 due to diagnostic challenges and, consequently, underreporting of non-O157 STEC. The low number of isolates and HUS cases prevented a serotype-specific statistical HUS association analysis. Furthermore, we only included STEC isolates in our study, and the prevalence of these virulence genes in other EPEC were not determined and, therefore, a direct association to the development of HUS is likely an overestimation. Also, we did not perform any gene expression analysis to confirm the level of gene translation. Lastly, data on host factors other than age were not available to allow for adjustment in our statistical model.

Our results showed that the non-LEE-encoded immune system modulator *nleH1–2*, together with age ≤5 years and *stx2a*, may contribute significantly in discerning HUS-associated STEC (PPV 73%). The OR of developing HUS from an STEC infection was eight times higher when stains were positive for *nleH1–2*. Larger studies are required to increase the statistical power of the reported significant associations to enable a better identification of HUS-associated STEC and review infection control guidelines in light of new knowledge. We recommend the Norwegian NRL to include screening for *nleH1–2* in routine STEC surveillance to improve the supervision of appropriate infection control measures for sporadic cases and during STEC outbreaks.

## Electronic supplementary material

Below are the links to the electronic supplementary material.

Fig. 2 Phylogenetic estimation of the relatedness of STEC isolates (*n* = 340) based on the absence or presence of 33 virulence genes and subtypes of *stx1* and *stx2*. Phylogenetic tree constructed using Dice similarity matrix with a complete linkage cluster analysis method with 100 bootstrap simulations. Isolates of major serotypes are coloured according to the legend. The presence of virulence genes is depicted by a black box under the corresponding virulence gene column. Isolates from cases developing HUS are highlighted with an orange box. (PDF 218 kb)

Table 3 (DOCX 67 kb)
