# Supplementary figures and images for: Virulence factors of Shiga toxin-producing Escherichia coli and the risk of developing haemolytic uraemic syndrome in Norway, 1992–2013
Source: Eur J Clin Microbiol Infect Dis. 2017 Apr 8;36(9):1613–20. doi: 10.1007/s10096-017-2974-z (PMC5554284; doi:10.1007/s10096-017-2974-z)

- NSFO157
- O103:H2
- O26:H11
- O145:H28
- SFO157
- O117:H7
- O145:H?
- O103:H25
- O111:H8

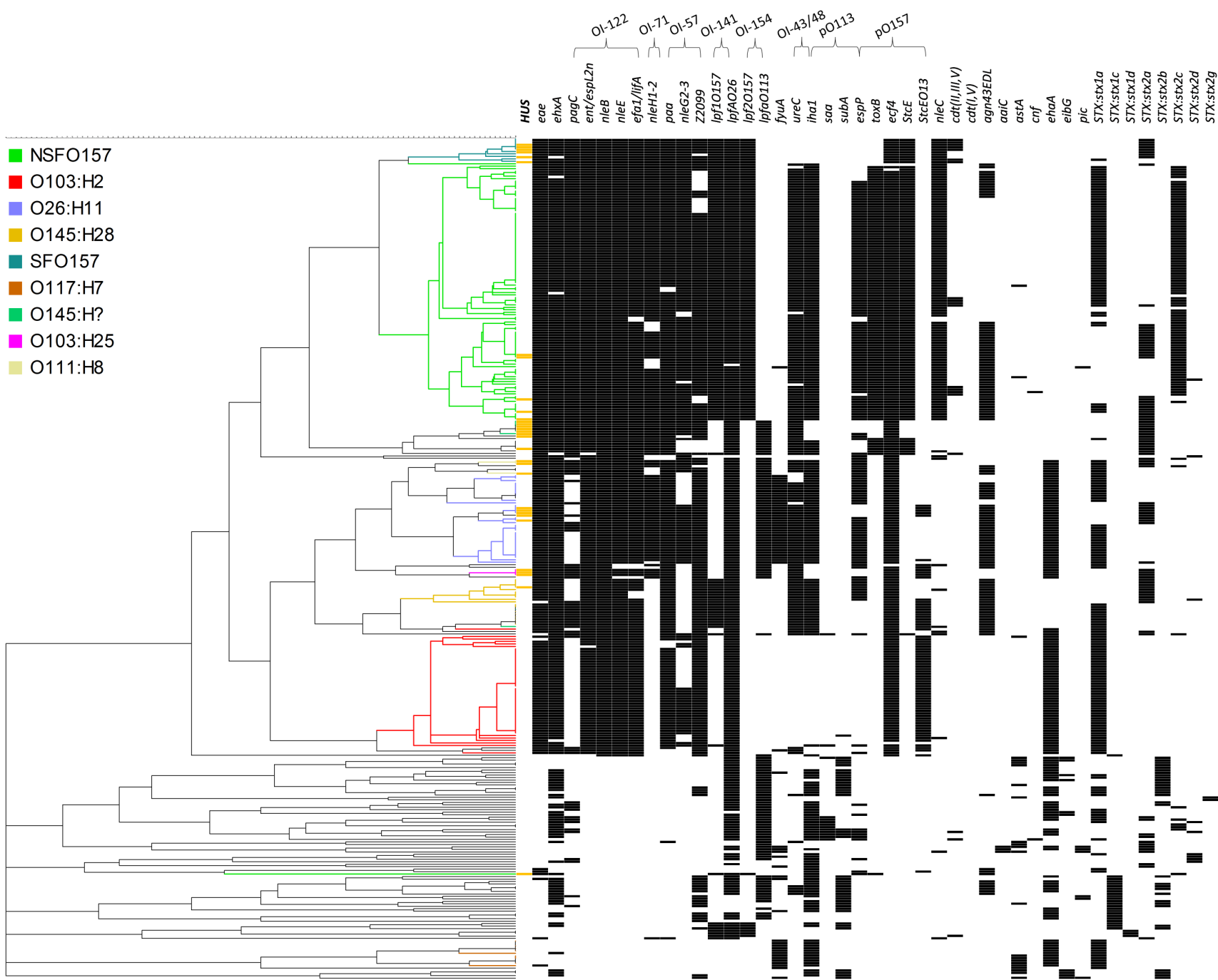

Supplement: Supplementary file 1 — Phylogenetic estimation of the relatedness of STEC isolates (n = 340) based on the absence or presence of 33 virulence genes and subtypes of stx1 and stx2. Phylogenetic tree constructed using Dice similarity matrix with a complete linkage cluster analysis method with 100 bootstrap simulations. Isolates of major serotypes are coloured according to the legend. The presence of virulence genes is depicted by a black box under the corresponding virulence gene column. Isolates from cases developing HUS are highlighted with an orange box. (PDF 218 kb) [file 10096_2017_2974_MOESM1_ESM.pdf]
