# Supplementary material for: Virulence factors of Shiga toxin-producing Escherichia coli and the risk of developing haemolytic uraemic syndrome in Norway, 1992–2013
Source: Eur J Clin Microbiol Infect Dis. 2017 Apr 8;36(9):1613–20. doi: 10.1007/s10096-017-2974-z (PMC5554284; doi:10.1007/s10096-017-2974-z)
Supplement: Supplementary file 2 — (DOCX 67 kb) [file 10096_2017_2974_MOESM2_ESM.docx]

Table 3 (Supplemental) Thirty-three virulence gene targets and corresponding primers used in three multiplex PCR setups for the screening of all STEC isolates (n=340) in this study.

| **Virulence Factors** | ***Genes*** | **Primer name** | **Multiplex PCR** | **Lable** | **Primer sequences (5'-3')** | **Amplicon (bp)** | **Reference** |
| --- | --- | --- | --- | --- | --- | --- | --- |
| Cytotoxic necrotizing factors 1, 2 and 3 | *cnf1-cnf2-cnf3* | cnf-univ-2-F | M1 | 6-FAM | AAACGYAGTTTYAGTGATGG | 221 | This study |
|  |  | cnf-univ-2-R |  |  | GAACGACGTTCTTCATAAGT |  |  |
| EAggEC heat-stable enterotoxin | *eastA* | astA | M1 | VIC | CCATCAACACAGTATATCCGA | 111 | [1] |
|  |  | astA |  |  | GGTCGCGAGTGACGGCTTTGT |  |  |
| Subtilase cytotoxin | *subA* | subA-LTB-F | M1 | 6-FAM | GTCAGYYAGGGCGGAAAAACC | 507 | This study |
|  |  | subA-LTB-R |  |  | GCGCCATCATTACCCACTGC |  |  |
| Secreted protease of C1 esterase inhibitor from EHEC (O157) | *StcE* | StcE-F | M1 | PET | AGCCCGCGATGATAATAATAAAAT | 419 | [2] |
|  |  | StcE-R |  |  | CGGAGCGGAACCACTGAC |  |  |
| Secreted protease of C1 esterase inhibitor from EHEC (O103) | *StcE_O103_* | StcEO103-F | M1 | VIC | ACCGGATTCAGCAAGTGG | 366 | [2] |
|  |  | StcEO103-R |  |  | CGTTTCGCGGGTATTCA |  |  |
| Cytolethal distending toxins | *cdtB (II, III, V)* | CDT-1F | M1 | VIC | GAAAGTAAATGGAATATAAATGTCCG | 466 | [3] |
|  |  | CDT-1R |  |  | AAATCACCAAGAATCATCCAGTTA |  |  |
| Cytolethal distending toxins | *cdtB (I, IV)* | CDT-2F | M1 | 6-FAM | GAAAATAAATGGAACACACATGTCCG | 466 | [3] |
|  |  | CDT-2R |  |  | AAATCTCCTGCAATCATCCAGTTA |  |  |
| STEC autoagglutinating adhesin | *saa* | saa-F | M2 | VIC | CGTGATGAACAGGCTATTGC | 119 | [4] |
|  |  | saa-R |  |  | ATGGACATGCCTGTGGCAAC |  |  |
| Novel nonhemagglutinin adhesin (from O157:H7 and CFT073) | *ihaA* | ihaA-F | M2 | 6-FAM | ACGCTGAAAGGCGGTGTGACCA | 178 | This study |
|  |  | ihaA-R |  |  | TCAGACCGGCGGGGTTATCGTAA |  |  |
| Phase-variable surface protein Ag43 | *agn43EDL933 (cah)* | agn43EDL933-F | M2 | VIC | CGCTGTGCCCGATAACTC | 240 | This study |
|  |  | agn43EDL933-R |  |  | GTGCCGGGACTGATGTGA |  |  |
| Long polar fimbria 1 (O157, OI141) | *lpfO157/OI-141/lpf1* | lpf1-F | M2 | PET | CTGCGCATTGCCGTAAC | 412 | [5] |
|  |  | lpf1-R |  |  | ATTTACAGGCGAGATCGTG |  |  |
| Long polar fimbria A (O26) | *lpfAO26* | lpfAO26-F | M2 | VIC | GTTCTGTTTGCCTTATCTGC | 509 | [5] |
|  |  | lpfAO26-R |  |  | TAAGTCAGGTTGAAGTCGAC |  |  |
| Long polar fimbria 2 (O157, OI154) | *lpfO157/OI-154/lpf2* | lpf2-F | M2 | 6-FAM | GCAGGTCACCTACAGGCGGC | 528 | [5] |
|  |  | lpf2-R |  |  | CTGCGAGTCGGCGTTAGCTG |  |  |
| Long polar fimbria A (O113) | *lpfAO113* | lpfAO113-1F | M2 | 6-FAM | GCATTCACTCTGGCATCTCTA | 498 | This study |
|  |  | lpfAO113-1R |  |  | CGTTACGGTCGCATTGG |  |  |
| *E.coli* immunoglobulin-binding (Eib) protein G | *eibG* | eibG-F | M2 | PET | ATCGGCTTTCATCGCATCAGGAC | 548 | [6] |
|  |  | eibG-R |  |  | CCACAAGGCGGGTATTCGTATC |  |  |
| Toxin B of *Clostridium difficile* | *toxB* | ToxB-F | M2 | VIC | TTGGGCGCATAGGTGAAG | 442 | This study |
|  |  | ToxB-R |  |  | CGCAAGGCCAACGGATTA |  |  |
| Extracellular serine protease P | *espP* | EspP-F | M2 | 6-FAM | AAACAGCAGGCACTTGAACG | 307 | [7] |
|  |  | EspP-R |  |  | AGACAGTTCCAGCGACAACC |  |  |
| EHEC autotransporter | *ehaA* | ehaA-F | M2 | PET | GACGGGTGAGCAGAAACAAACC | 503 | [8] |
|  |  | ehaA-R |  |  | ATCCAGGTAACCTGTGCTTGCG |  |  |
| EAEC-specific type VI secretion system | *aaiC* | aaiC-F | M2 | PET | ATTGTCCTCAGGCATTTCAC | 215 | [9] |
|  |  | aaiC-R |  |  | ACGACACCCCTGATAAACAA |  |  |
| Pesticin receptor | *fyuA* | fyuA-F1 | M3 | VIC | ATGTACCCGCGCCACCCAGGAC | 416 | This study |
|  |  | fyuA-R1 |  |  | TGTATGCGGCCAGCGTTTCAGC |  |  |
| Actin microfilament aggregation inducer | *ent/espL2n* | sen-F1 | M3 | VIC | TACTCCTGGATTATTTTCTGC | 456 | This study |
|  |  | sen-R1 |  |  | TATTCCTTTACTGCCTTATTTGA |  |  |
| non-LEE-encoded protein B | *nleB* | nleB-F | M3 | 6-FAM | GGAAGTTTGTTTACAGAGACG | 297 | [10] |
|  |  | nleB-R |  |  | AAAATGCCGCTTGATACC |  |  |
| non-LEE-encoded protein E | *nleE* | nleE-F | M3 | PET | CTAATACTCAGGGCGTGTCC | 192 | [11] |
|  |  | nleE-R |  |  | ACCGTCTGGCTTTCTCGTTA |  |  |
| EHEC factor for adherence | *efa1/lifA* | efa1tot-F | M3 | 6-FAM | CAGGTAACTGGAAATCATCATTG | 104 | [12] |
|  |  | efa1tot-R |  |  | ATCTCTGTTGAAAGTGTTAGGG |  |  |
| PagC | *pagC like* | pagC-F1 | M3 | 6-FAM | TACGCTATCGGCTGGTTA | 448 | This study |
|  |  | pagC-R1 |  |  | ATCTGCTTTAGTTCCCTCATA |  |  |
| non-LEE-encoded protein H1-2 | *nleH1-2* | nleH1-2-F | M3 | 6-FAM | ATGTTATCGCCCTCTTCTAT | 413 | This study |
|  |  | nleH1-2-R |  |  | CTAACCCTCGGCAACTCTGATT |  |  |
| Urease subunit alpha | *ureC* | ureC-F | M3 | PET | TCTAACGCCACAACCTGTAC | 398 | [13, 14] |
|  |  | ureC-R |  |  | GAGGAAGGCAGAATATTGGG |  |  |
| non-LEE-encoded protein C | *nleC* | nleC-F | M3 | PET | CGCCCCGGCAGGATACTC | 464 | This study |
|  |  | nleC-R |  |  | CTGGCGCAACACTAAAACTGACAT |  |  |
| Lipid A myristoyl transferase | *ecf4* | ecf4-F | M3 | VIC | TGAGATAAGCAGGCAGATACC | 130 | [12] |
|  |  | ecf4-R |  |  | AAACACACAGATAATACCGATACC |  |  |
| Porcine attaching and effacing associated factor | *paa* | paa-F | M3 | VIC | ATGAGGAACATAATGGCAGG | 360 | [14] |
|  |  | paa-R |  |  | TCTGGTCAGGTCGTCAATAC |  |  |
| EAEC Mucinase | *pic* | pic-F | M3 | 6-FAM | ACTGGATCTTAAGGCTCAGGAT | 567 | [14, 15] |
|  |  | pic-R |  |  | GACTTAATGTCACTGTTCAGCG |  |  |
| Ubiquitin ligase | *nleG2-3* | nleG2-3-F | M3 | PET | ATGGGCGCGGAGATATTACAGTC | 422 | This study |
|  |  | nleG2-3-R |  |  | TATTGGTTCCCGGGTCAGTGG |  |  |
| Hypotehtical protein (EDL933) | *Z2099* | Z2099-F | M3 | 6-FAM | GTAGCGCAGGCAAAAGAACT | 141 | This study |
|  |  | Z2099-R |  |  | GGCCCATACGCTGTCATCAA |  |  |

REFERENCES

1. Nakazawa, T., K. Agematsu, and A. Yabuhara, *Later development of Fas ligand-mediated cytotoxicity as compared with granule-mediated cytotoxicity during the maturation of natural killer cells.* Immunology, 1997. **92**(2): p. 180-7.

2. Brandal, L.T., et al., *First report of the Shiga toxin 1 gene in sorbitol-fermenting Escherichia coli O157:H(-).* J Clin Microbiol, 2012. **50**(5): p. 1825-6.

3. Toth, I., et al., *Production of cytolethal distending toxins by pathogenic Escherichia coli strains isolated from human and animal sources: establishment of the existence of a new cdt variant (Type IV).* J Clin Microbiol, 2003. **41**(9): p. 4285-91.

4. Paton, A.W. and J.C. Paton, *Direct detection and characterization of Shiga toxigenic Escherichia coli by multiplex PCR for stx1, stx2, eae, ehxA, and saa.* J Clin Microbiol, 2002. **40**(1): p. 271-4.

5. Toma, C., et al., *The long polar fimbriae genes identified in Shiga toxin-producing Escherichia coli are present in other diarrheagenic E. coli and in the standard E. coli collection of reference (ECOR) strains.* Res Microbiol, 2006. **157**(2): p. 153-61.

6. Merkel, V., et al., *Distribution and phylogeny of immunoglobulin-binding protein G in Shiga toxin-producing Escherichia coli and its association with adherence phenotypes.* Infect Immun, 2010. **78**(8): p. 3625-36.

7. McNally, A., et al., *Differences in levels of secreted locus of enterocyte effacement proteins between human disease-associated and bovine Escherichia coli O157.* Infect Immun, 2005. **73**(4): p. 2571.

8. Wells, T.J., et al., *EhaA is a novel autotransporter protein of enterohemorrhagic Escherichia coli O157:H7 that contributes to adhesion and biofilm formation.* Environ Microbiol, 2008. **10**(3): p. 589-604.

9. Boisen, N., et al., *New adhesin of enteroaggregative Escherichia coli related to the Afa/Dr/AAF family.* Infect Immun, 2008. **76**(7): p. 3281-92.

10. Wickham, M.E., et al., *Bacterial genetic determinants of non-O157 STEC outbreaks and hemolytic-uremic syndrome after infection.* J Infect Dis, 2006. **194**(6): p. 819-27.

11. Afset, J.E., et al., *Identification of virulence genes linked with diarrhea due to atypical enteropathogenic Escherichia coli by DNA microarray analysis and PCR.* J Clin Microbiol, 2006. **44**(10): p. 3703-11.

12. Andersson, T., et al., *Modeling gene associations for virulence classification of verocytotoxin-producing E. coli (VTEC) from patients and beef.* Virulence, 2011. **2**(1): p. 41-53.

13. Nakano, M., et al., *Association of the urease gene with enterohemorrhagic Escherichia coli strains irrespective of their serogroups.* J Clin Microbiol, 2001. **39**(12): p. 4541-3.

14. Brandt, S.M., et al., *Molecular risk assessment and epidemiological typing of Shiga toxin-producing Escherichia coli by using a novel PCR binary typing system.* Appl Environ Microbiol, 2011. **77**(7): p. 2458-70.

15. Henderson, I.R., et al., *Characterization of pic, a secreted protease of Shigella flexneri and enteroaggregative Escherichia coli.* Infection and immunity, 1999. **67**(11): p. 5587-96.
